# Personality and Party Ideology Among Politicians. A Closer Look at Political Elites From Canada and Belgium

**DOI:** 10.3389/fpsyg.2018.00552

**Published:** 2018-04-17

**Authors:** Jeroen K. Joly, Joeri Hofmans, Peter Loewen

**Affiliations:** ^1^Department of Political Science, Ghent University, Ghent, Belgium; ^2^Department of Work and Organizational Psychology, Vrije Universiteit Brussel, Brussels, Belgium; ^3^Department of Political Science, University of Toronto, Toronto, ON, Canada

**Keywords:** personality, Big Five, political ideology, political elites, politics

## Abstract

We examined the relationship between Big Five personality and the political ideology of elected politicians. To this end, we studied 303 politicians from Flanders, Wallonia, and Canada, relating their self-reported Big Five scores to a partisanship-based measure of political ideology. Our findings show that, in line with the congruency model of personality, Openness to Experience is the best and most consistent correlate of political ideology, with politicians high on Openness to Experience being more likely to be found among the more progressive left-wing political parties.

## Introduction

The political choices we make have a strong impact on our everyday life. As elected politicians are expected to express views and pursue goals that benefit society, their job is to pass laws, to decide on the increase or decrease of taxes, and to decide on ways to organize society. Given the enormous impact of political choices, it is not surprising that there is an increased interest in the study of political behavior, including its relationship with personality. Until today, however, most studies on the relationship between political behavior and personality have focused on the general population, showing for example that personality relates to political ideology (van Hiel et al., 2000), political attitudes (Gerber et al., 2010), ideological voting behavior (Vecchione et al., 2011), and political participation (Vecchione and Caprara, 2009; Mondak, 2010).

What is largely missing, however, are studies that focus on the personality of elected officials. This gap is notable, as elected officials are the actors who are given formal powers and thus great latitude in guiding societies. Moreover, the few studies that have studied politicians have demonstrated that there are important differences between the general population and elected officials, with the latter group for example scoring higher on Extraversion and Agreeableness than the general population (Caprara et al., 2003; Gerber et al., 2011). These initial findings suggest that findings from general populations cannot be assumed to straightforwardly transfer to the population of politicians (Caprara et al., 2003, 2010; Best, 2011). In the present study, we address this issue by explicitly focusing on the relationship between personality and political ideology in a sample of elected politicians.

The second important contribution of our study is that we study political ideology using a partisanship-based measure, namely the Chapel Hill Expert Study (CHES) scores. These party-related ideology scores are obtained by averaging ideological left-right scores assigned to each political party by country experts. An important advantage to measuring political ideology using the CHES scores is that, although partisanship-based political ideology is arguably only an approximation of a politician’s true ideology, it is an objective measure that is not susceptible to self-report response biases. Indeed, the large majority of studies on the association between personality and political ideology measures political ideology using self-reported ideology scores (see the meta-analysis by Sibley et al., 2012). This is potentially problematic, as self-reports are susceptible to various biases, such as social desirability bias (on which politicians are known to score higher than non-politicians; see Caprara et al., 2003), individual differences in (the lack of) introspective ability, and a wide range of rating scale biases. Because in these studies personality is typically also measured using self-reports (for an exception, see Carney et al., 2008), the relationships between personality and political ideology might be due to common method variance—or spurious covariance attributable to the measurement method rather than to the construct the measure represents (Podsakoff et al., 2003). By using partisanship-based political ideology scores, we ascertain that the relationships between personality and political ideology in our study are not caused by common method variance.

Third, we study the relationship between personality and partisanship-based political ideology in three political systems (i.e., Flanders, Wallonia, and Canada), each with their own unique constellation of political parties. This is an important contribution as previous research has predominantly relied on data from a single political system, while the relationships between personality and political ideology – examined among the general population – have been shown to differ between political systems (e.g., van Hiel et al., 2000; Thorisdottir et al., 2007; Roets et al., 2014). Hence, our approach allows testing to what extent the associations between personality and partisanship-based political ideology are specific for one system or generalizable across political systems.

### The Relationship Between Personality and Partisanship-Based Political Ideology

Political systems are generally divided alongside a number of cleavages or political dimensions that map onto a classic ideological divide between left and right (Lipset and Rokkan, 1967). The principal cleavage constituting this ideological continuum is economic, with left-wing politicians being more in favor of state intervention into the economy than right-wing politicians who prefer free market dynamics (Aarts et al., 2014). A second, main cleavage is the social one, where left-wing politicians are more tolerant of new life-styles and right-wing politicians promote more traditional arrangements. At the individual level, several studies found that the social/cultural and economic dimensions are distinct (Duckitt et al., 2002; Feldman and Johnston, 2014), although economic and social forms of conservatism were both found to be similarly associated with right-wing orientation (Napier and Jost, 2008)^1^.

In contrast to research at the individual level, research on party positions has revealed that, whereas the variation in political ideology between individuals can best be captured by two dimensions, the variation between parties can generally be explained by a single principal component — interpreted as the left-right divide (McDonald and Budge, 2005; Bara and Weale, 2006; Benoit and Laver, 2006). Because we measure political ideology using the CHES scores, being a partisanship-based measure of ideology, we draw on this one-dimensional left-right conceptualization of political ideology. Note that such a one-dimensional conceptualization is in line with the conceptualization of political ideology in previous studies that used party-based measures of ideology, such as studies using party identification or voting behavior (van Hiel et al., 2000; Jost, 2006; Schoen and Schumann, 2007; Caprara et al., 2010; Vecchione et al., 2011).

Previous research on the relationship between personality and political ideology has shown that, among the general population, Openness to Experience and Conscientiousness are consistently the best personality predictors of political ideology (see Sibley et al., 2012 for a meta-analysis). These findings are explained by the congruency model of Caprara and Zimbardo (2004), which holds that people endorse ideologies that are in line with their own personality traits and values. Importantly, such a congruency principle not only operates for political attitudes and behaviors, but holds for attitudes and behavior in general. That is, research in a wide variety of applied settings has shown that one important way through which personality “gets outside the skin” is by selecting situations that allow for the expression of one’s personality traits (Hampson, 2012; Frederickx and Hofmans, 2014; Judge et al., 2017).

Applied to the relationship between Openness to Experiences and political ideology, the congruency model implies that, because people high on Openness to Experiences are typically open to a wide variety of experiences, are more tolerant toward others, other ways of living and people with different value systems, they are expected to be more inclined to hold left-wing political ideology, which often involves acceptance of unconventional behaviors and economic proposals entailing the involvement of the government in the economy (Gerber et al., 2011). In support of this idea, previous research has found that Openness to Experiences predicts political orientation among the general population across a wide range of countries (van Hiel et al., 2000; Caprara et al., 2006, 2010; Jost, 2006; Schoen and Schumann, 2007; Vecchione et al., 2011), while the relationship has also been observed among elected officials in Italy (Caprara et al., 2010).

Note that the expected relationship between Openness to Experiences and partisanship-based political ideology is also in line with research on the relationship between Right Wing Authoritarianism (RWA) and the Big Five. RWA, being a blend of submissiveness toward authorities, aggressiveness against outgroups and people who deviate from the norm, and adherence to traditions and norms, has been shown to predict social policy support and political party support (Duckitt et al., 2010). Among the Big Five, it is best predicted by Openness to Experiences, with people low on Openness to Experiences scoring high on RWA (Sibley and Duckitt, 2008). In sum, research on the general population supports the relationship between Openness to Experiences and political ideology. Drawing on the congruency principle, we expect this relationship to also hold for elected politicians. That is, we expect politicians who score high on Openness to Experiences to be more likely to belong to parties characterized by left-wing, rather than right-wing political ideology (Hypothesis 1).

Regarding Conscientiousness, being the tendency for impulse control and achievement striving, and the predisposition to follow rules and norms, to plan and to organize (John and Srivastava, 1999), high scorers are expected to more often hold right-wing, rather than left-wing political ideology. This expectation is fully in line with the congruency principle, as right-wing political ideology is related to order, closure, structure, and decisiveness, which are features that align well with the specific facets characterizing Conscientiousness (Carney et al., 2008). Empirically, this relationship has been supported by a weak yet significant meta-analytical correlation between Conscientiousness and political ideology in the general population (Sibley et al., 2012), a relationship with a preference for right rather than left-wing parties among the general population (Vecchione et al., 2011), and a link between Conscientiousness and support for conservative parties and candidates (Gerber et al., 2011). Based on these research findings in the general population, and drawing on the congruency principle, we expect that politicians scoring high on Conscientiousness will more likely belong to parties characterized by right-wing, rather than left-wing political ideology (Hypothesis 2).

With respect to the other Big Five traits, we have no explicit expectations. The reason is that the left-right distinction is less relevant to Extraversion, Agreeableness and Emotional Stability, which concern energetic approach in the social and material world, prosocial and communal orientation toward others, and even-temperedness, respectively (John and Srivastava, 1999). In line with this reasoning, meta-analytic research has shown that, among the Big Five, the associations between Extraversion, Agreeableness and Emotional Stability and political orientation were “(at best) trivial” (Sibley et al., 2012, p. 672), while also those with RWA were “negligible” (Sibley and Duckitt, 2008, p. 257).

## Materials and Methods

### Participants and Procedure

Flemish, Walloon, and Canadian politicians were contacted to participate in a study on information processing and political representation. These politicians came from different levels of government. Because party systems are defined through the parties that present themselves to voters during elections, we look at Flanders and Wallonia separately, but do not distinguish between representatives from Canada and Ontario, where the party systems overlap almost perfectly. **Table 1** displays the descriptive statistics for our different party systems.

**Table 1:** Descriptive sample statistics.

	*N*	Age		Gender	Mean tenure	Gov. Level	
				
		*M*	*SD*	*Male*	*Years*	*Federal*	*Regional*
Canada	71	53.4	11.7	64%	8.2	45	26
Flanders	156	44.8	9.1	64%	7.1	62	94
Wallonia	76	49.2	10.6	68%	7.1	33	43


Politicians were contacted by a local team of experts. While most politicians were approached through their staff, Flemish Belgian Members of Parliament (MP) were contacted directly by the team leader, which partly explains differences in response rate (65,8% in Belgium – 79% for Flemish and 49% for francophone politicians – and 27,3% in Canada). MPs were repeatedly contacted by the local research teams until a definitive yes/no answer was obtained. This insistence was necessary, given the low response rates generally found among political elite surveys (Hoffman-Lange, 2008; Bailer, 2014; Deschouwer and Depauw, 2014; Walgrave and Joly, 2017) for a variety of reasons, including lack of time or the reluctance to divulge personal information and participate in a standardized and ‘impersonal’ survey.

Participants took part in an extended interview/survey on political representation and information-processing. At the beginning of the interview/survey, respondents were informed—both verbally and written—that their participation was anonymous and their answers were strictly confidential. Most questions in the interview/survey pertained to their day-to-day work as a representative, probing their attention to recent events in the news, their policy priorities, as well as those of the public, and which information sources they relied on for their most important political activities, etc. The interview part was built around the standardized survey questions, asking for more detailed information, examples or clarification. As the last part of the survey, once rapport and trust had been established, politicians were presented with the Ten-Item Personality Inventory (TIPI), a self-report personality questionnaire (Gosling et al., 2003). The entire meeting took approximately one hour on average.

### Measures

#### Big Five Personality

To assess politicians’ personality, we used the TIPI. This short instrument offers a rapid way to measure individuals’ personalities (Gosling et al., 2003). In our study, we used the revised Dutch version of Hofmans et al. (2008) for Flemish politicians and the French version developed by Erica Carlisle and revised by Mike Friedman for the Walloon politicians (Friedman, n.d.).

**Table 2** presents the average scores and standard deviations per Big Five trait per political party system, as well as the Spearman–Brown internal consistency indices—which are preferred over Cronbach alpha for scales with few items (Eisinga et al., 2013).

**Table 2:** Means, standard deviations, and Spearman–Brown reliability index per Big Five dimension and per political party system.

	Canada			Flanders			Wallonia		
			
	*M*	*SD*	*S–B*	*M*	*SD*	*S–B*	*M*	*SD*	*S–B*
Openness	5.55	0.94	0.38	5.26	0.98	0.84	5.13	0.98	0.62
Conscientiousness	5.66	1.08	0.77	5.09	1.00	0.28	5.88	0.91	0.26
Extraversion	4.77	1.70	0.93	4.98	1.27	0.97	4.98	1.21	0.34
Agreeableness	5.14	1.07	0.53	4.93	0.89	0.00	5.05	0.90	0.00
Emotional Stability	5.54	1.05	0.84	5.30	1.03	0.81	5.10	1.27	0.70


#### Partisanship-Based Ideology

Politicians’ ideology was measured through partisan affiliation using the CHES data (Polk et al., 2017). To construct its ideology scale, CHES relies on national experts from each country, who score each party in terms of its ideological stance between 0 (Extreme left) and 10 (Extreme right). The mean of these left-right ideological scores across experts constitutes the ideological position for each party. Comparison of different indicators of ideological party positions revealed expert scores to be among the most valid (Ray, 2007).

## Results

### Bivariate Correlations

Pearson correlations between CHES ideology and the Big Five personality traits are shown in **Table 3**. Because the relationships between CHES ideology and the Big Five personality traits were studied in three political systems (i.e., Flanders, Wallonia, and Canada), each with their own unique constellation of political parties, we first tested the correlations for each political system separately. In line with Hypothesis 1, we found that Openness related negatively to CHES ideology in Canada and Flanders, showing that having a more right-wing ideology is associated with lower scores on Openness. In contradiction to Hypothesis 2, however, no relationship was found between Conscientiousness and CHES ideology. Moreover, for the other Big Five traits, no relationship with CHES ideology was found.

**Table 3:** Pearson correlations between Big Five personality traits and ideological position Chapel Hill Expert Study (CHES).

	*Full sample*	*Canada*	*Flanders*	*Wallonia*
Openness	-0.14 ^∗∗^	-0.23^∗^	-0.15^∗^	-0.10
Conscientiousness	-0.09	-0.00	0.07	-0.07
Extraversion	0.05	0.13	0.03	-0.06
Agreeableness	-0.06	-0.17	-0.01	0.06
Emotional Stability	-0.03	-0.07	-0.07	0.04


*^∗∗∗^*p* < 0.01, ^∗∗^*p* < 0.05, ^∗^*p* < 0.1.*

Subsequently, we tested whether the strength of the correlations differed between political systems using a test of the equality of correlations based on Fisher’s Z-transformation (Cohen and Cohen, 1983). Only one of the 15 comparisons (i.e., 3 comparisons per Big Five trait) approached conventional levels of significance (i.e., for agreeableness the difference in correlation coefficients between Canada (*r* = -0.17) and Wallonia (*r* = 0.06) had a *p*-value of 0.081). Therefore, we merged the data for the three political systems and also performed a correlation analysis on the data of the full sample (the advantage of this analysis being that it has more statistical power than the separate analyses). This analysis confirmed our previous findings, showing that CHES ideology is negatively related to Openness, whereas it is unrelated to the other Big Five traits (see **Table 3**).

### Regression Analyses

Subsequently, we regressed CHES ideology on all Big Five traits simultaneously (see **Table 4**). In all regressions, we controlled for age and gender, as these variables have been shown to relate to left-right preferences in previous studies (e.g., Inglehart and Norris, 2000; Poggione, 2004). Similar to the correlational analyses, we first regressed CHES ideology on the Big Five traits for each political system separately. Overall, the results were in line with the correlational analyses, revealing a negative relationship between Openness to Experience and CHES ideology in Canada and Flanders but not in Wallonia, while the relationship between Conscientiousness and CHES ideology was not statistically significant. Hence, Hypothesis 1 was accepted, while we found no support for Hypothesis 2. Moreover, unlike the correlational analyses, we found higher levels of extraversion to be associated with more right-wing party affiliation in Canada, but not in Flanders and Wallonia.

**Table 4:** OLS regression analysis explaining ideological position (CHES).

	*Full sample*			*Canada*			*Flanders*			*Wallonia*		
				
	*B*	β	*t*	*B*	β	*t*	*B*	β	*t*	*B*	β	*t*
Openness	-0.35	-0.16	-2.92***	-0.53	-0.25	-2.24**	-0.35	-0.16	-1.92*	-0.19	-0.09	-0.78
Conscientiousness	0.08	0.04	0.67	0.13	0.07	0.60	0.21	0.10	1.19	-0.23	-0.11	-0.81
Extraversion	0.17	0.11	1.86*	0.26	0.22	1.86*	0.13	0.08	0.87	0.12	0.07	0.56
Agreeableness	0.02	0.01	0.15	-0.13	-0.07	-0.60	0.11	-0.08	0.52	0.14	0.07	0.54
Emotional stability	-0.08	-0.04	-0.66	-0.09	-0.05	-0.38	-0.16	0.05	-0.84	0.04	0.02	0.18
Canada	-01.19	-0.23	-3.86***	-	-	-	-	-	-	-	-	-
wallonia	-1.77	-0.36	-5.97***	-	-	-	-	-	-	-	-	-
Gender (1 = female)	-0.71	-0.16	-2.74***	-1.24	-0.29	-2.10**	-0.48	-0.11	-1.31	-0.64	-0.16	-1.24
Age	0.01	0.05	0.83	0.02	0.10	0.76	-0.02	-0.08	-0.97	0.04	0.20	1.60
Intercept	6.78		5.32***	6.46		2.48**	7.49		4.10***	3.58		

Observations	303			71			156			76		
R-squared	0.17			0.22			0.05			0.09		
Adjusted R-squared	0.15			0.14			0.01			-0.02		


*^∗∗∗^*p* < 0.01, ^∗∗^*p* < 0.05, ^∗^*p* < 0.1.*

Next, we tested whether there were differences between the three political systems in the extent to which the Big Five traits predicted CHES ideology using a test for the equality of regression coefficients (Paternoster et al., 1998). None of the 15 comparisons (i.e., 3 comparisons per Big Five trait) was statistically significant (i.e., all *p*-values > 00.15), suggesting that we can safely merge the data and perform a regression analysis on the full sample. The results of this final analysis were in line with the findings of our individual analyses, showing that CHES ideology was negatively predicted by Openness and positively by Extraversion.

## Discussion

This study examined the link between personality and political ideology among elected politicians. Looking at partisan affiliation of politicians in three different party systems, we found Openness to Experience to consistently correlate with political ideology in our general sample of elected politicians and in two out of our three political party systems. Since people who are high on Openness to Experience are typically more tolerant toward others, toward other ways of living and toward people with different value systems, these people are more likely to be found among the more progressive left-wing political parties. This finding can be explained by the congruency model of Caprara and Zimbardo (2004), holding that people endorse ideologies that are in line with their own personality traits. Moreover, this result replicates the findings of previous research, showing that Openness to Experience is the most important and systematic predictor of politicians’ party affiliation (Caprara et al., 2010) and the best personality predictor of party preference among the general population (Vecchione et al., 2011). It is also in line with the negative relationship between Openness to Experience and RWA (Sibley and Duckitt, 2008).

The hypothesized relationship between Conscientiousness and political ideology was not confirmed in our study. Although one might expect this to be due to range restriction in Conscientiousness scores among politicians – who are believed to score highly on Conscientiousness – this explanation is contradicted by both previous research and our own data. In particular, previous studies comparing politicians and the general population have shown that, despite the straightforward laymen belief that politicians are highly conscientious, both groups do not differ regarding Conscientiousness (e.g., Caprara et al., 2003). Moreover, also in our own sample, the average Conscientiousness scores and the standard deviations for the politicians were similar to the scores for a comparable sample of voters. An alternative explanation for the lack of a correlation with Conscientiousness might be that especially the facets order and dutifulness are relevant for political ideology in the sense that these facets align most with order, closure, structure, and decisiveness, which all characterize right-wing ideology. However, in the TIPI measure, only one adjective (i.e., disorganized) taps into these facets, while the other three adjectives pertain to the facets self-discipline, competence, and deliberation (the remaining adjectives are self-disciplined, dependable, and careless), which are clearly less relevant when it comes to congruency with right-wing ideology. For this reason, we believe that our data are not conclusive, and that the relationship between politician’s Conscientiousness and political ideology is in need of further research. In future studies, it might be interesting to not only test the effect of broad Big Five traits, but also look at narrow facets, drawing on the idea that the relationship with political ideology might be driven by a small subset of Conscientiousness facets only.

An important element of our study is that we measured political ideology through partisan membership. This operationalization of political ideology has a number of distinctive advantages. First, partisan membership is not susceptible to response biases, such as socially desirable answering, which is known to be higher in politicians as compared to the general population (Caprara et al., 2003). Second, because personality is self-rated while political ideology is measured using objective, behavioral data, the relationship between Openness to Experience and political ideology cannot be a methodological artifact caused by same-source bias. Third, by looking at expert ratings of political ideology based on partisan membership, we differentiate along the whole left-right continuum, rather than merely classifying politicians in center-left or center-right politicians, as previous studies have done (e.g., Caprara et al., 2003). Besides its distinctive advantages, there are also some drawbacks associated with measuring political ideology through partisan membership. First, it neglects the fact that, within a party, there are differences in the ideological positions between politicians sharing the same ideology. Second, the decision to join a particular political party is not exclusively influenced by the political ideology of the politician, but also by a number of other factors, like the probability of being elected as a candidate for a given party, the expected possibilities of influencing policy within that party, or the presence of existing networks within the party. The consequence of this is that partisan membership is only a rough proxy of political ideology, which means that the associations found in the present study are probably underestimations of the true associations. Therefore, future research with other indicators of political ideology (e.g., behavioral measures, like roll call voting or other-ratings of the politician) is needed.

Because elected politicians are a hard to reach group with little time (Hoffman-Lange, 2008; Bailer, 2014; Deschouwer and Depauw, 2014), our individual sample sizes are rather modest. Nevertheless, the total sample size of 303 observations is decent, offering sufficient statistical power (>0.75) for the detection of small to moderate correlations. A related concern is that the response rates in Wallonia, and particularly in Canada, were modest at best. Despite these modest response rates, however, we obtained a representative sample of the political parties in each political system. Moreover, despite large differences in response rates, we observed little difference between the associations for the three political systems.

A second implication of the time constrains related to studying elected politicians was that we had to use a very brief 10-item Big Five measure. Although previous research shows that TIPI scores converge with standard, multi-item Big Five scores, predict external correlates, and reach adequate levels of test-retest reliability (Gosling et al., 2003), its internal consistency indices are typically low. The reason is that, having only two items per Big Five dimension, TIPI “*emphasized content validity considerations, resulting in lower inter-item correlations than is typical of more homogeneous scales*” (Gosling et al., 2003, p. 516). Such low internal consistency indices are also observed in our study. Whereas this is not problematic because internal consistency is not a goal in itself, but only matters to the extent that it serves validity (Hofmans et al., in press), we nevertheless tested whether low internal consistency suppressed some of the personality-ideology relationships. To this end, we checked the correlations between ideology and the individual TIPI items. Although a small number of significant correlations were found, they were highly sample-specific. Therefore, we do not believe that the low internal consistencies undermine our general finding that across party systems, Openness to Experience is the best personality predictor of political ideology.

To sum up, Openness to Experience was found to correlate to partisan-based political ideology, with politicians scoring high on Openness to Experience being more likely to be found in progressive left-wing political parties. This is an interesting finding because it aligns with meta-analytic research on the general population showing that also in non-politicians, Openness to Experience is the best personality predictor of political conservatism (Sibley et al., 2012). Despite these clear parallels, the association is not very strong, suggesting that the lion’s share of variance in partisan-based political ideology is explained by other factors beyond personality. A case in point is that, unlike in the general population (Sibley et al., 2012), no relationship with Conscientiousness was observed. Whereas this was unexpected, it underscores our claim that findings from non-politicians cannot be assumed to straightforwardly transfer to the population of politicians (see also Caprara et al., 2003, 2010; Best, 2011).

## Ethics Statement

This study was carried out in accordance with the recommendations of the Research Ethics Board (REB) of the University of Toronto and the Ethische Adviescommissie Sociale en Humane Wetenschappen (EA SHW) of the University of Antwerp with written informed consent from all subjects in accordance with the Declaration of Helsinki. The protocol was approved by the Research Ethics Board (REB) of the University of Toronto and the Ethische Adviescommissie Sociale en Humane Wetenschappen (EA SHW) of the University of Antwerp.

## Author Contributions

JJ and PL took part in the initial conception of the project and the data collection. JJ and JH carried out the analyses. All the authors co-wrote the final paper.

## Conflict of Interest Statement

The authors declare that the research was conducted in the absence of any commercial or financial relationships that could be construed as a potential conflict of interest.

## Footnotes

1 For a comprehensive review of the conceptual structure and functions of political ideology, see Jost et al. (2009).
